# Occupational Exposure to Needle Stick and Sharp Injuries and Associated Factors among Health Care Workers in Awi Zone, Amhara Regional State, Northwest Ethiopia, 2016

**DOI:** 10.1155/2017/2438713

**Published:** 2017-08-10

**Authors:** Abebe Dilie, Desalegn Amare, Tenaw Gualu

**Affiliations:** ^1^Department of Nursing, College of Health Sciences, Debre Markos University, Debre Markos, Ethiopia; ^2^Department of Nursing, College of Health Sciences, Bahir Dar University, Bahir Dar, Ethiopia

## Abstract

**Background:**

Needle stick and sharp injuries were one of the major risk factors for blood and body fluid borne infections at health care facilities.

**Objective:**

To assess occupational exposure to needle stick and sharp injuries and associated factors among health care workers in Awi zone, 2016.

**Methods:**

institutional based cross-sectional study was conducted among 193 health care workers. Study participants were selected using systematic random sampling technique.

**Result:**

When queried, 18.7% of the respondents' encountered needle stick and sharp injury in the last 1 year. Participants who practiced needle recapping and had job related stress were 21.3 and 7.3 times more likely to face needle stick and sharp injury, respectively. However, those who apply universal precautions and acquire the required skill were 99% and 96% times less likely to face needle stick and sharp injury, respectively, than their counterparts.

**Conclusion and Recommendation:**

The prevalence of needle stick and sharp injury was relatively low as compared to previous studies. Recapping of needle after use, job related stress, not applying universal precautions, and lack of the required skill were associated with needle stick and sharp injuries. Therefore, health care providers should apply universal precaution.

## 1. Introduction

Every year, hundreds of thousands of health care workers are exposed to dangerous and deadly blood borne pathogens through contaminated needle stick and sharps injuries (NSIs) because of performing daily procedures in clinical activities. These exposures can carry the risk of infection with with Hepatitis B (HBV), Hepatitis C (HCV), and Human Immunodeficiency Virus (HIV), the virus that causes AIDS [1].

The risk of infection following needle stick exposure is 1.9% to greater than 40% for HBV infections, 2.7% to 10% for HCV infections, and 0.2% to 0.44% for HIV infections. It is estimated that NSIs cause approximately 66,000 HBV infections, 16,000 HCV infections, and 200 to 5000 HIV infections among health care workers annually. However, postexposure management was generally poor [2].

A study in in Bahir Dar, Amhara, Ethiopia, showed that 65.9% of health care providers were exposed to blood and body fluids in the past year, of which 29.0% were needle stick injuries [3]. NSIs can be regarded as preventable, if health care workers adopt a comprehensive program that addresses institutional, behavioral, and device-related factors that contribute to the occurrence of needle stick injuries in health care workers [4].

A quasi-experimental study concludes that occupational exposure to blood borne pathogens can be prevented through improved knowledge and behavior and reduced the number of NSI among health care providers [5].

A study indicated that the highest incidence of NSIs was seen in health care providers and that the associated factors were age, level of education, number of shifts per month, and history of related training. The highest rate of NSIs was related to instrument preparation, injection, and recapping of used needles [6].

NSIs are frequent and important cause of morbidity and mortality in health care workers who come into contact with patient blood and body fluids [7]. Therefore, assessing the magnitude and factors associated with needle stick and sharp injury among health care workers is very important.

## 2. Methods and Materials

An institutional based cross-sectional study was conducted from April to May, 2016, to assess occupational exposure of NSI among health care providers in Awi zone.

All health care providers who work both at private and public health institutions in Awi zone who were willing to participate and available during study period were included in the study. However, health care providers who were in annual leave and seriously ill during data collection period within the zone were excluded from the study.

The sample size was determined based on single population proportion formula with 5% marginal error and 95% confidence interval by considering 42% proportion prevalence of NSI among health care workers from Arba Minch, southern Ethiopia [8]. Besides this, by considering correction formula and 10% nonresponse rate, a total of 213 health care workers were included in this study.

Study participants were selected using systematic random sampling after proportional allocation was done for each professional category in hospitals and health centers by including all districts with in the zone.

The outcome variable of the study was occupational exposure to needle stick and sharp injury. The independent variables of the study were sociodemographic factors (sex, age, religion, marital status, level of education, monthly salary, and work experience), work environment related factors (health and safety information access, work shift, availability of safety box, sharp disposal, application of universal precautions, needle recap, and working with assistance during procedure), and behavioral related factors (substance use, sleeping disorder, job satisfaction, job related stress, and perceived skill acquisition).

Data were collected using structured self-administered questionnaire. The questionnaire was adapted by reviewing literatures of similar studies on needle stick and sharp injury [1, 9]. The data were collected by 4 trained diploma nurses and were supervised by 2 public health professionals having previous experience in data collection. Continuous follow-up and supervision were also made by principal investigator throughout the data collection period.

Data quality was assured by training of data collectors and supervisors, pretesting the questionnaire on similar setting (10% of total sample) that was not included in the study, close supervision and assistance of data collectors, and checking filled questionnaires on daily basis for completeness, clarity, and accuracy of data.

The data were entered in to EPI-data version 3.1, and then the data were cleaned and analyzed by using Statistical Package for Social Science (SPSS) version 21 statistical software. Bivariate and multivariate logistic regression was computed to assess statistical association between the outcome variable and independent variables using odds ratio; significance of statistical association was assured or tested using 95% confidence interval (CI) and *p* value (<0.05).

The following terms were described in such a way in this study.

*(i) Needle Sticks and Sharp Injury (NSI).* It is any kind of injury which occurred on the health care worker in relation to his/her job in the health institution within 1-month period.

*(ii) Severe NSI.* It is deep stick/cut or profuse bleeding related to injury.

*(iii) Moderate NSI.* It is skin punctured and some bleeding associated with injury.

*(iv) Superficial NSI.* It is little or no bleeding related to injury.

*(v) Perceived Skill.* It is the acquisition of required skill to perform procedures as reported by the health care provider.

The study was conducted after obtaining ethical clearance from Debre Markos University, College of Health Sciences, Research and Ethical Review Committee. After obtaining official letter from the college, a permission and support letter were provided to Awi zone hospitals and health centers before data collection. The study participants were informed about the objective, rationale, and expected outcomes of the study and written consent was provided for guaranteeing their choice of participation or refusal. All the information was recorded anonymously and confidentiality was assured throughout the study.

## 3. Results

### 3.1. Sociodemographic Characteristics

A total of 213 eligible health workers were included in the study. Of these, only 193 health workers voluntarily agreed to participate in this study, and 20 either refused or submitted largely incomplete questionnaires. This resulted in a response rate of 90.6%.

Of all participants, the majority, 172 (89.1%), were orthodox Christian by religion and three-fourths (75%) of the respondents had ≤5 years' work experience. Moreover, the age of the participants included in this study ranged between 22 and 48 years with mean age of 28.25 (SD = ±6.149) years (Table 1).

### 3.2. Needle Stick and Sharp Injury (NSI)

Among the respondents, 36 (18.7%) encountered NSI in the last 1 year. Of these, 28 (77.8%) were due to needles, 2 (5.6%) were due to surgical equipment, and 6 (16.6%) were due to medication ampoule or vial. About 86% of NSI occurred when performing injection (Table 2).

### 3.3. Work Environment Related Factors for NSI

Among the total respondents, 172 (89.1%) apply universal precautions during procedures for protection and 30 (15.5%) of the respondents had reported that there are needles and sharp materials outside safety box (Table 3).

### 3.4. Behavioral Risk Factors of Health Care Workers

Out of the respondents, 53 (27.6%) believed NSI is nonpreventable and 33 (17.1%) used substance. Moreover, 118 (61.1%) were satisfied with their job and 97 (50.3%) had sleep disturbance (Table 4).

### 3.5. Factors Associated with Needle Stick and Sharp Injuries

In bivariate logistic regression analysis, sex of the respondent, profession, information access to health and safety, application of universal precaution, recapping of needle, perceived skill acquisition, and job related stress were statistically associated with NSI with *p* value less than 0.05 at 95% confidence interval (Table 5).

After bivariate analysis, only those variables which were significantly related (*p* value < 0.05) were entered for further multivariate analysis. By adjusting potential confounders in multivariate logistic regression analysis, only sex, application of universal precaution, recapping of needle, perceived skill acquisition, and job related stress were significantly associated with NSI. But participant's profession and information access to health and safety were not significantly associated with NSI in multivariate analysis.

Application of universal precaution and perceived skill acquisition were negatively associated with NSI. Health care providers who use universal precautions were 99% times less likely to face NSI [AOR = 0.01 (0.002, 0.1)] as compared to those who did not use universal precaution. Moreover, health workers who had acquired the required skill were 96% times less likely to encounter NSI [AOR = 0.04 (0.003, 0.57)] than those who did not have the required skill.

Similarly, male health workers were 10 times more likely to encounter NSI [AOR = 10 (1.5, 16)] than females. Health workers who had job related stress were 7.3 times more likely to face NSI [AOR = 7.3 (1.6, 33.2)] than those who did not have job related stress. Moreover, health workers who recap needles were 21.3 times more likely to encounter NSI [AOR = 21.3 (4.4, 23)] than those who did not recap needle.

## 4. Discussion

The study showed that 18.7% of the respondents had needle stick and sharp injury at least once in the previous 1 year. This finding is lower than a study done in Ondo state, Nigeria, where 55.8% of health care providers encounter needle stick and sharp injury [10]. This might be due to the presence of trainings and safety guidelines that advocate for proper patient and self-care as well as study time differences. However, it is relatively similar with a study done in Bale zone, Ethiopia, where 19.1% had needle stick and sharp injury [11]. This might be due to having relatively similar sociodemographic characteristics.

The most important factor that affects needle stick and sharp injury was application of universal precautions during procedure. Health care providers who use universal precaution were 99% times less likely to face needle stick and sharp injuries [AOR = 0.01 (0.002, 0.1)] as compared to those who did not use universal precaution. This might be due to the fact that applying universal precautions can act as a barrier from exposure to blood and body fluid.

Another factor that affects needle stick and sharp injury in this study was recapping of needle after use. Health workers who recap needles were 21.3 times more likely to face needle stick and sharp injury [AOR = 21.3 (4.4, 23)] than those who did not recap needle. A similar study done in Arba Minch also showed having significant association between NSI and recapping of needle [AOR = 4.2 (1.5, 12.0)] [8]. This might be due to increased risk of injury when recapping needle after use.

In this study, male health workers were 10 times more likely to face needle stick and sharp injury (AOR = 10 (1.5, 6.6)) than females. This is relatively similar with a study done at Ondo state Nigeria, where male workers are likely to sustain more NSIs than female workers (OR = [1.987 (1.061–3.721)]) [10]. This may be due to the fact that females are better in safety precautions compared to males.

Moreover, health workers who had perceived skill acquisition were 96% times less likely to encounter needle and sharp injury [AOR = 0.04 (0.003, 0.57)] than those who did not have the required skill. Another study which was in United States also showed that lack of skill accounts for 12% occurrence of needle stick injury [12]. This might be due to the fact that not knowing when and how to apply the required skill can also expose professionals at risk of NSI.

In addition, health workers who had job related stress were 7.3 times more likely to face needle stick and sharp injury (AOR = 7.3 (1.6, 33.2)) than those who did not have job related stress. However, this contradicts a study done at Felege Hiwot Referral Hospital, where those who were satisfied on their job were about 3 times more likely to sustain needle stick and sharp injury than those who were not satisfied on their job [AOR = 2.78 (1.01, 7.63)] [13].

## 5. Strengths and Limitations of the Study

### 5.1. Strength of the Study

The major strength of this study lies in the fact that it has attempted to assess occupational exposure to NSI and associated factors for the first time in the study area. Besides this, both private and public health institutions within the zone were included to make the study representative.

### 5.2. Limitation of the Study

The study might be subjected to response set bias from the respondent. Since it was cross-sectional study design, it was difficult to draw causal relationships.

We exclude seriously ill participants as they may suffer from needle stick and sharp acquired blood borne pathogens.

## 6. Conclusion and Recommendation

The prevalence of needle stick and sharp injury was relatively low as compared to previous studies.

The most important factors that cause needle stick and sharp injury were recapping of needle after use, job related stress, not using universal precautions during procedure, and lack of the required skill. Safe handling and disposal of needle stick and sharp materials enable preventing blood borne infections. Therefore, health care providers should get training to fill the skill gap, apply universal precaution during procedure, and never recap needles after use.

## Figures and Tables

**Table 1:** Sociodemographic characteristics of health care workers in Awi zone, 2016.

Variable	Frequency (%)
*Sex *	
Male	106 (54.9)
Female	87 (45.1)
*Age *	
20–29 years	149 (77.2)
30–39 years	29 (15)
≥40 years	15 (7.8)
*Religion *	
Orthodox	172 (89.1)
Muslim	13 (6.7)
Protestant	8 (4.2)
*Marital status *	
Single	103 (53.4)
Married	90 (46.6)
*Educational level*	
Diploma	95 (49.2)
Degree	83 (43)
Masters	13 (6.7)
Specialty	2 (1)
*Monthly income (in Ethiopian Birr)*	
<3000	116 (60.1)
3001–4999	22 (11.4)
≥5000	55 (28.5)
*Service year *	
0–5 years	145 (75.1)
6–10 years	33 (17.1)
≥11 years	15 (7.8)
*Profession *	
Nursing	76 (39.4)
Public health	32 (16.6)
Midwifery	49 (25.4)
Laboratory	24 (12.4)
Physician	12 (6.2)

**Table 2:** NSI among health care providers in Awi zone, 2016.

NSI	Frequency (%)

*Did you encounter NSI?*	
Yes	36 (18.7)
No	157 (81.3)
*Which device caused the injury?*	
Needles	28 (77.8)
Surgical equipment^*∗*^	2 (5.6)
Medication ampoule/vial	6 (16.6)
*Task performed during injury*	
Suturing	2 (5.5)
Injection	31 (86)
Drawing sample	2 (5.5)
Recap of needle	1 (3)
*Type of injury*	
Severe^†^	8 (22.2)
Moderate^*✠*^	9 (25)
Superficial^★^	19 (52.8)
*Was source patient identifiable?*	
Yes	15 (41.6)
No	21 (58.4)
*Did you receive care after injury?*	
Yes	23 (63.8)
No	13 (36.2)

^*∗*^Lancet, suturing needle, scalpel, and towel clip. ^†^Deep stick/cut or profuse bleeding. ^*✠*^Skin punctured, some bleeding. ^★^Little or no bleeding.

**Table 3:** Health care providers working environment condition in Awi zone, 2016.

Working environment risk factors	Frequency (%)
*Working institution *	
Hospital	83 (43)
Health center	110 (57)
*Shift work *	
Yes	66 (34.2)
No	127 (65.8)
*Information access to safety *	
Yes	185 (95.9)
No	8 (4.1)
*Use of universal precaution *	
Yes	172 (89.1)
No	21 (10.9)
*Did you recap needles?*	
Yes	49 (25.4)
No	144 (74.6)
*Availability of safety box *	
Yes	189 (97.9)
No	4 (2.1)
*Presence of needles and sharp materials outside safety box*	
Yes	30 (15.5)
No	163 (84.5)
*Condition of safety box*	
Overfilled	164 (85)
Torn out	13 (6.7)
Empty	12 (6.2)
Others	4 (2.1)
*Sharp waste final disposal*	
Burn and burry	35 (18.1)
Incinerator	150 (77.7)
Open dumping	8 (4.2)

**Table 4:** Behavioral characteristics of health care workers in Awi zone, 2016.

Behavioral risk factor	Frequency (%)
*Sleep disturbance *	
Yes	97 (50.3)
No	96 (49.7)
*Perceived skill acquisition *	
Yes	65 (33.7)
No	128 (66.3)
*Doing with assistance*	
Yes	19 (9.8)
No	174 (90.2)
*Substance use *	
Yes	33 (17.1)
No	160 (82.9)
*Job satisfaction *	
Yes	118 (61.1)
No	75 (38.9)
*Job related stress*	
Yes	24 (12.4)
No	169 (87.6)
*Belief on the risky nature of NSI *	
High risk	100 (51.8)
Moderate risk	88 (45.6)
Low risk	5 (2.6)
*Belief on preventability of NSI*	
Preventable	140 (72.5)
Nonpreventable	53 (27.6)

**Table 5:** Bivariate and multivariate logistic regression analysis of factors associated with NSI in Awi zone, 2016.

Needle stick and sharp injury (NSI)						
Variable	Response	Yes	No	COR(95% CI)^*∗*^	AOR(95% CI)^*∗∗*^	*p* value
*Sex *	Male	27 (75%)	79 (50.3%)	2.9 (1.3, 6.7)	10 (1.5, 16)	**0.014**
	Female	9 (25%)	78 (49.7%)	1	1	
*Profession*	Nursing	11 (30.6%)	65 (41.4%)	5.9 (1.6, 21.6)	0.75 (0.09, 5.8)	0.79
	Public health	6 (16.7%)	26 (16.6%)	4.3 (1.1, 18.2)	0.43 (0.06, 2.9)	0.39
	Midwifery	9 (25%)	40 (25.5%)	4.4 (1.2, 17.1)	0.24 (0.01, 3.6)	0.30
	Laboratory	4 (11.1%)	20 (12.7%)	5 (1.0, 23.7)	0.17 (0.01, 1.5)	0.12
	Physician	6 (16.7%)	6 (3.8%)	1	1	
*Information access*	Yes	32 (88.9%)	153 (97.5%)	0.20 (0.5, 0.8)	0.25 (0.01, 5.4)	0.37
	No	4 (11.1%)	4 (2.5%)	1	1	
*Use universal precaution*	Yes	18 (50%)	154 (98.1%)	0.01 (0.005, 0.073)	0.01 (0.002, 0.1)	**0.001**
	No	18 (50%)	3 (1.9%)	1	1	
*Recap of needle*	Yes	22 (61.1%)	27 (17.2%)	7.5 (3.4, 16.6)	21.3 (4.4, 23)	**0.001**
	No	14 (38.9%)	130 (82.8%)	1	1	
*Perceived skill*	Yes	6 (16.7%)	59 (37.6%)	0.33 (0.1, 0.8)	0.04 (0.003, 0.57)	**0.017**
	No	30 (83.3%)	98 (62.4%)	1	1	
*Job related stress*	Yes	11 (30.6%)	13 (8.3%)	4.8 (1.9, 12)	7.3 (1.6, 33.2)	**0.010**
	No	25 (69.4%)	144 (91.7%)	1	1	

^*∗*^COR: crude odds ratio; ^*∗∗*^AOR: adjusted odds ratio; CI: confidence interval.
